# Existence of a Conserved Quantity and Stability of *In Vitro* Virus Infection Dynamics Models with Absorption Effect

**DOI:** 10.1155/2019/2954041

**Published:** 2019-03-03

**Authors:** Celia Martínez-Lázaro, Marco Antonio Taneco-Hernández, Ramón Reyes-Carreto, Cruz Vargas-De-León

**Affiliations:** ^1^Facultad de Matemáticas, Universidad Autónoma de Guerrero Chilpancingo, Av. Lázaro Cárdenas S/N, Cd. Universitaria, 39087 Chilpancingo, Guerrero, Mexico; ^2^Escuela Superior de Medicina, Instituto Politécnico Nacional, Plan de San Luis y Díaz Mirón S/N, Col. Casco de Santo Tomas, Del. Miguel Hidalgo, 11340 Ciudad de México, Mexico

## Abstract

The estimation of parameters in biomathematical models is useful to characterize quantitatively the dynamics of biological processes. In this paper, we consider some systems of ordinary differential equations (ODEs) modelling the viral dynamics in a cell culture. These models incorporate the loss of viral particles due to the absorption into target cells. We estimated the parameters of models by least-squares minimization between numerical solution of the system and experimental data of cell cultures. We derived a first integral or conserved quantity, and we proved the use of experimental data in order to test the conservation law. The systems have nonhyperbolic equilibrium points, and the conditions for their stability are obtained by using a Lyapunov function. We complemented these theoretical results with some numerical simulations.

## 1. Introduction

In mathematical biology models, two of the best-known conserved quantities correspond to the Lotka–Volterra predator-prey and Kermack–McKendrick SIR models. The conserved quantities are also known as first integrals associated with the systems of differential equations that describe the process or phenomenon of interest; particularly, in physics, there are several examples, namely, the Hamiltonian systems, among others. In biology, a conserved quantity can be used as a null hypothesis, compared to values from a real population, to describe statistically significant deviations from the first integral. In ecology, the Lotka–Volterra predator-prey system is used as a null model, and if the biomass is not conserved, it leads to look for factors that are affecting the habitat of the species. In epidemiology, the classical SIR epidemic model without vital dynamics has a first integral that is used to calculate the maximum number of infected individuals (*I*_max_) reached in the epidemic. In other words, the conserved quantity is used to predict when the number of infected individuals will begin to decline. It is important to find conserved quantities in mathematical models focused on biology because of their relevance.

Interest has recently increased in the development of methods to find first integrals of biological systems. Nucci and Sanchini [1] applied Lie group analysis to a two-dimensional population model and found that it can be integrated by quadrature under some conditions on the parameters. To derive the first integrals of a two-dimensional epidemic model with nonlinear relapse, Naz et al. [2] use the partial Lagrangian approach developed by Kara et al., in [3, 4]. Also, Naz [5] uses the partial Hamiltonian approach to derive another first integral of the classical Lotka–Volterra model that had not been reported in the literature. Pugliese and collaborators [6] find conserved equations for the epidemic multidimensional systems; among these, a model with heterosexual transmission and another model of Zika virus sexual transmission, which under certain conditions, allow to calculate the basic reproductive number *R*_0_ and determine the stability of the disease-free equilibrium state.

At the cellular level, a conserved quantity occurring in an *in vitro* experiment will indicate that a biological material (amino acids, nucleic acids, and even the materials used by a cell to preserve its viability and function) is constant. Kakizoe and collaborators in [7] reported the existence of a conserved quantity in a basic model of viral infection in a cell culture.

In the mathematical theory of *in vivo* and *in vitro* viral infections, the basic models are concentrated on population dynamics of target cells and the interaction between virus particles and target cells, but several of the *in vivo* or *in vitro* models ignore the absorption effect of the virus particle or viral genome. The viral dynamics that considers the absorption effect has been studied by the following authors. Perelson et al. studied a four-dimensional system for the interaction of HIV with CD4^+^ T cells and incorporated the absorption effect when a virus infects an uninfected cell [8]. Berreta and Kuang studied the dynamics of marine bacteriophages. They determined two equilibria, and obtained stability conditions for the infection-free and infected equilibrium states, and derived conditions for Hopf bifurcation to occur [9]. Smith and De Leenheer proposed a family of viral infection models with a generalized function of cell dynamics and effect of absorption. They determined the basic reproductive number and proved the global stability of infection equilibrium by using the theory of competitive systems [10]. Iggidr and collaborators [11] proposed a malaria intrahost model with *k* classes of age for the parasitized erythrocytes and *n* strains for the parasite. They calculated the basic reproductive number and proved the global stability of equilibria by using the Lyapunov function method. Beauchemin and collaborators [12] developed two influenza viral infection models *in vitro*, where they estimated the parameters of models based on in vitro virological data under various constant concentrations of amantadine. One of such models includes the absorption effect and a discrete intracellular delay. Furthermore, they also compared between the models with or without absorption.

Our first goal in this paper is to prove the existence a conserved quantity of *in vitro* virus infection dynamics models with absorption effect, as well as demonstrate the stability of the nonhyperbolic equilibrium points. Our second goal is to estimate the parameters and selection of one of the four models that can describe the experimental data of virus infection in cell cultures. Our last goal is to test the law of conservation experimentally.

The organization of this paper is as follows. In Section 2, we present the extension to two *in vitro* models, which consider the effect of loss of a viral particle, which is called the absorption effect when it infects uninfected cells. In Section 3, we found a first integral of each system, and we discuss and establish the conditions for the stability of each model by using the technology of Lyapunov functions. In Section 4, we estimate the parameters of models with and without absorption effect by least-squares minimization between numerical solution of the system and experimental data, respectively. For the selection between the models, we use the Akaike information criterion (AIC_C_) [13], which tells us which model is relatively better. In Section 5, we demonstrate the use of experimental data in order to test the conservation law. In Section 6, we use a biologically realistic range of parameter values to present some numerical simulations. Lastly, in Section 7, we provide a few concluding remarks.

## 2. An *In Vitro* Viral Infection Model with Absorption Effect

The model

(1) $$\begin{array}{c} \dot{x}\left(t\right)=-\beta x\left(t\right)v\left(t\right),\\ \dot{y}\left(t\right)=\beta x\left(t\right)v\left(t\right)-\delta y\left(t\right),\\ \dot{v}\left(t\right)=py\left(t\right)-cv\left(t\right), \end{array}$$

is a modified version of the basic virus dynamics model [14, 15]; this system was proposed in 2008 by Beauchemin et al. [12]. *x*(*t*) and *y*(*t*) are the numbers of target (susceptible) and infected (virus-producing) cells per ml of medium, respectively, and *v*(*t*) is the viral load per ml of the medium.

The parameters are such that *β* is the rate at which virions infect the target cells, *δ* is the rate of death of infected cells, *p* is the production rate of infectious virions by infected cells, and *c* is the virion clearance rate.

Recently, the authors [16, 17] reduced system (1) to a two-dimensional SIR-type model under the assumption that the viral dynamics is much faster than the infected cell dynamics and that a quasi-stationary state at which *v*=*py*/*c* is attained very quickly. In particular, the authors applied the classical results derived from the SIR epidemic model to the context of the dynamic viral infections, and they calculated the area under the viral load curve, initial viral growth rate, peak viral load, and time to peak viral load, among other quantities.

**Figure 1:**
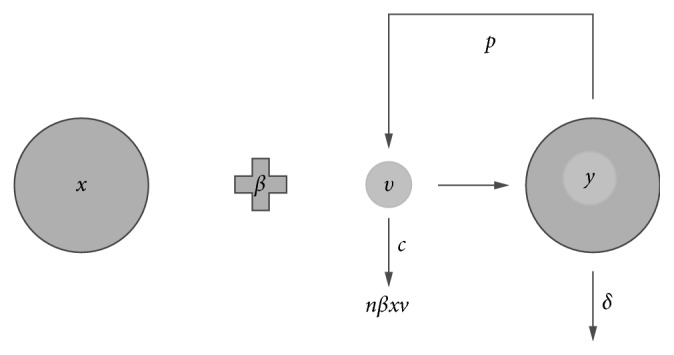
A schematic diagram of the *in vitro* viral infection model with absorption effect.

The process of absorption of a viral particle or its genome is one of the first steps of a viral infection. This process has been modeled by a lot of authors [9–11, 18, 19] and ignored by many others. We introduce in system (1) the absorption effect, which is modeled by incorporating a bilinear term *nβx*(*t*)*v*(*t*) in the third equation, where 0 < *n* < *p*/*δ*. The parameter *n* is the average number of viral particles (or their viral genome) that enters a cell (see Figure 1). Consequently, the extended system is given by the following systems of differential equations:

(2) $$\begin{array}{c} \dot{x}\left(t\right)=-\beta x\left(t\right)v\left(t\right),\\ \dot{y}\left(t\right)=\beta x\left(t\right)v\left(t\right)-\delta y\left(t\right),\\ \dot{v}\left(t\right)=py\left(t\right)-cv\left(t\right)-n\beta x\left(t\right)v\left(t\right). \end{array}$$

The biological feasible region of system (2) is given as

(3) $$\begin{array}{c} \Omega ≔\left\{\left(x,y,v\right)\in {ℜ}^{3}:x\geq 0,y\geq 0,v\geq 0\right\}. \end{array}$$

Another alternative model is the one based on the effect of the latent phase:

(4) $$\begin{array}{c} \dot{x}\left(t\right)=-\beta x\left(t\right)v\left(t\right),\\ \dot{w}\left(t\right)=\beta x\left(t\right)v\left(t\right)-\phi w\left(t\right),\\ \dot{y}\left(t\right)=\phi w\left(t\right)-\delta y\left(t\right),\\ \dot{v}\left(t\right)=py\left(t\right)-cv\left(t\right)-n\beta x\left(t\right)v\left(t\right), \end{array}$$

where *w*(*t*) is the number of infected cells in the latent phase and the parameter *ϕ* is the rate at which infected cells in the latent phase become productively infected cells. Put another way, 1/*ϕ* is the period of time that elapses between the viral entry and the transcription of viral RNA. The other parameters are the same as in the previous models.

The biological feasible region of the system (4) is given as

(5) $$\begin{array}{c} \Omega ≔\left\{\left(x,w,y,v\right)\in {ℜ}^{4}:x\geq 0,w\geq 0,y\geq 0,v\geq 0\right\}. \end{array}$$

When *n*=0, it corresponds to systems (2) and (4) without absorption effect.

## 3. Properties of Models

### 3.1. First Integral

In the following, we prove the existence of a first integral of systems (2) and (4). The idea of constructing the expressions of the first integrals consists of combining known forms (Volterra-type and linear functions) with a kind of undetermined coefficients technique.

**Theorem 1 .**

System (2) has the following first integral:

(6) $$\begin{array}{c} H\left(x\left(t\right),y\left(t\right),v\left(t\right)\right)=x\left(t\right)-\frac{c\delta }{\beta \left(p-n\delta \right)}-\frac{c\delta }{\beta \left(p-n\delta \right)}\ln\;x\left(t\right)+\frac{p}{p-n\delta }y\left(t\right)+\frac{\delta }{p-n\delta }v\left(t\right). \end{array}$$

**Proof**

Computing the derivative of (12) along the solutions of system (2), we obtain

(7) $$\begin{array}{c} \frac{d}{dt}H\left(x,y,v\right)=\left(1-\frac{c\delta }{\beta \left(p-n\delta \right)}\frac{1}{x}\right)\left[-\beta xv\right]+\frac{p}{p-n\delta }\left[\beta xv-\delta y\right]+\frac{\delta }{p-n\delta }\left[py-cv-n\beta xv\right]\\ =-\beta xv+\frac{p\beta }{p-n\delta }xv-\frac{n\beta \delta }{p-n\delta }xv\\ =\beta \left[-1-\frac{n\delta }{p-n\delta }+\frac{p}{p-n\delta }\right]xv=0. \end{array}$$

The proof is complete from which the following is obtained.

**Corollary 1 .**

System (2) has the following normalized first integral:

(8) $$\begin{array}{c} Q\left(x\left(t\right),y\left(t\right),v\left(t\right)\right)=\frac{p}{\left(p-n\delta \right)x\left(0\right)}\left(\left(1-\frac{n\delta }{p}\right)x\left(t\right)+\frac{c\delta }{p}\int_{0}^{t}v\left(\tau \right)d\tau +y\left(t\right)+\frac{\delta }{p}\left(v\left(t\right)-v\left(0\right)\right)\right)=1. \end{array}$$

**Proof**

A time integration renders the conserved quantity *H*(*x*(*t*), *y*(*t*), *v*(*t*)) ≡ *H*(*x*(0), *y*(0), *v*(0)) ≡ *C*:

(9) $$\begin{array}{c} x\left(t\right)-\frac{c\delta }{\beta \left(p-n\delta \right)}\ln\;x\left(t\right)+\frac{p}{p-n\delta }y\left(t\right)+\frac{\delta }{p-n\delta }v\left(t\right)\equiv x\left(0\right)-\frac{c\delta }{\beta \left(p-n\delta \right)}\ln\;x\left(0\right)+\frac{p}{p-n\delta }y\left(0\right)+\frac{\delta }{p-n\delta }v\left(0\right). \end{array}$$

In a cell culture, the initial condition of the infected cells is *y*(0)=0. We have

(10) $$\begin{array}{c} \left(1-\frac{n\delta }{p}\right)x\left(t\right)-\frac{c\delta }{p\beta }\ln\frac{x\left(t\right)}{x\left(0\right)}+y\left(t\right)+\frac{\delta }{p}\left(v\left(t\right)-v\left(0\right)\right)=\left(1-\frac{n\delta }{p}\right)x\left(0\right). \end{array}$$

From the first equation of system (2), we get the following expression ln(*x*(*t*)/*x*(0))=−*β*∫_0_^*t*^*v*(*τ*)*dτ*. We have

(11) $$\begin{array}{c} Q\left(x\left(t\right),y\left(t\right),v\left(t\right)\right)=\frac{p}{\left(p-n\delta \right)x\left(0\right)}\left[\left(1-\frac{n\delta }{p}\right)x\left(t\right)+\frac{c\delta }{p}\int_{0}^{t}v\left(\tau \right)d\tau +y\left(t\right)+\frac{\delta }{p}\left(v\left(t\right)-v\left(0\right)\right)\right]=1. \end{array}$$

Next, we will enunciate and we will demonstrate results similar to the previous ones but this time for system (4). In the following theorem, we prove the existence of a first integral of system (4).

**Theorem 2 .**

System (4) has the following first integral:

(12) $$\begin{array}{c} H\left(x\left(t\right),w\left(t\right),y\left(t\right),v\left(t\right)\right)=x\left(t\right)-\frac{c\delta }{\beta \left(p-n\delta \right)}-\frac{c\delta }{\beta \left(p-n\delta \right)}\ln\;x\left(t\right)+\frac{p}{p-n\delta }\left(w\left(t\right)+y\left(t\right)\right)+\frac{\delta }{p-n\delta }v\left(t\right). \end{array}$$

The proof is essentially the same as the previous theorem, except with obvious modifications in the construction of the *H* function.

**Corollary 2 .**

System (2) has the following normalized first integral:

(13) $$\begin{array}{c} Q\left(x\left(t\right),w\left(t\right),y\left(t\right),v\left(t\right)\right)=\frac{p}{\left(p-n\delta \right)x\left(0\right)}\left[\left(1-\frac{n\delta }{p}\right)x\left(t\right)+\frac{c\delta }{p}\int_{0}^{t}v\left(\tau \right)d\tau +w\left(t\right)+y\left(t\right)+\frac{\delta }{p}\left(v\left(t\right)-v\left(0\right)\right)\right]=1. \end{array}$$

**Remark 1 .**

The following terms are present in the first integral:

- *p*/*δ*, viral burst size.
- *v*(*t*) − *v*(0), viral load difference between time *t* and time zero.
- ∫_0_^*t*^*v*(*τ*)*dτ*, the area under the viral load curve.
- *x*(*t*)=*x*(0)exp(−*β*∫_0_^*t*^*v*(*τ*)*dτ*), the number of target cells at time *t*.

These quantities are important as they are biologically interpreted and they provide information on viral dynamics.

The *H*(*t*) and *Q*(*t*) quantities are interpreted as follows:

“The total biological material (amino acids, nucleic acids, and other biomolecules) is preserved from the start to end of the viral infection.”

### 3.2. Stability of Equilibria

To analyze systems (2) and (4) and discuss the stability, we first find out the equilibrium points. Let

(14) $$\begin{array}{c} \dot{x}\left(t\right)=0,\\ \dot{y}\left(t\right)=0,\\ \dot{v}\left(t\right)=0. \end{array}$$

After calculating, system (2) has two equilibrium points: *p*_0_=(0,0,0) and a line (continuum) of infection-free equilibria *p*_1_=(*x*^*∗*^, 0,0), with *x*^*∗*^ > 0.

Similarly, from system (4), let

(15) $$\begin{array}{c} \dot{x}\left(t\right)=0,\\ \dot{w}\left(t\right)=0,\\ \dot{y}\left(t\right)=0,\\ \dot{v}\left(t\right)=0. \end{array}$$

So, after calculating, system (4) has two equilibrium points: *p*_0_=(0,0,0,0) and a line (continuum) of infection-free equilibria *p*_1_=(*x*^*∗*^, 0,0,0), with *x*^*∗*^ > 0.

We note that the equilibrium points for two systems are nonhyperbolic. For this reason, we will study the stability of these equilibria by using the Lyapunov function method.

We construct a linear Lyapunov function for the stability analysis of the trivial equilibrium point for system (2).

**Theorem 3 .**

The trivial equilibrium point *p*_0_=(0,0,0) of system (2) is stable.

**Proof**

The Lyapunov function is defined as follows:

(16) $$\begin{array}{c} W\left(x,y,v\right)=3x\left(t\right)+2y\left(t\right)+\frac{\delta }{p}v\left(t\right). \end{array}$$

The *W* function is positive definite on Ω, and clearly *W*(0,0,0)=0. The time derivative of *W* along the solution of (2) is given by

(17) $$\begin{array}{c} \dot{W}\left(x,y,v\right)=3\dot{x}\left(t\right)+2\dot{y}\left(t\right)+\frac{\delta }{p}\dot{v}\left(t\right)\\ =-3\beta x\left(t\right)v\left(t\right)+2\left(\beta x\left(t\right)v\left(t\right)-\delta y\left(t\right)\right)+\frac{\delta }{p}\left(py\left(t\right)-cv\left(t\right)-n\beta x\left(t\right)v\left(t\right)\right)\\ =-\left(\beta x\left(t\right)v\left(t\right)+\delta y\left(t\right)+\frac{c\delta }{p}v\left(t\right)+\frac{n\delta \beta }{p}x\left(t\right)v\left(t\right)\right)<0. \end{array}$$

Therefore, by using Lyapunov's theorem, the *p*_0_ trivial equilibrium point is stable.

We obtain similar results for system (4). In this case, using the linear Lyapunov function *W*(*x*, *w*, *y*, *v*)=3*x*(*t*)+2(*w*(*t*)+*y*(*t*))+(*δ*/*p*)*v*(*t*) and Lyapunov's theorem, we have the following theorem.

**Theorem 4 .**

The trivial equilibrium point *p*_0_=(0,0,0,0) of system (4) is stable.

In the following, we consider the stability of any infection-free equilibrium point of line (continuum) of equilibria *p*_1_=(*x*^*∗*^, 0,0) with *x*^*∗*^ > 0 for system (2). We construct a Lyapunov function for infection-free equilibrium, using suitable combinations of common quadratic and linear functions.

**Theorem 5 .**

Assume that $\hat{R}=\left(\beta \left(p-n\delta \right)/c\delta \right)x^{*}$. If $\hat{R}<1$ and *p* > *nδ*, then any infection-free equilibrium point *p*_1_=(*x*^*∗*^, 0,0) of line (continuum) of equilibria of system (2) is stable on Ω.

**Proof**

The Lyapunov function is defined as

(18) $$\begin{array}{c} L\left(x,y,v\right)≔\frac{1}{2x^{*}}{\left(x-x^{*}\right)}^{2}+\frac{p\beta }{c\delta }x^{*}y+\frac{\beta }{c}x^{*}v. \end{array}$$

The *L* function is positive definite for *x*, *y*, *z* ∈  Ω, and *L*(*x*^*∗*^, 0,0)=0. The time derivative of *L* computed along solutions of (2) is

(19) $$\begin{array}{c} \dot{L}\left(x,y,v\right)=\frac{1}{x^{*}}\left(x-x^{*}\right)\dot{x}+\frac{p\beta }{c\delta }x^{*}\dot{y}+\frac{\beta }{c}x^{*}\dot{v}\\ =-\frac{\beta v}{x^{*}}{\left(x-x^{*}\right)}^{2}-\left(1-\frac{\beta \left(p-n\delta \right)}{c\delta }x^{*}\right)\beta xv\\ =-\beta v\left(\frac{1}{x^{*}}{\left(x-x^{*}\right)}^{2}+\left(1-\hat{R}\right)x\right). \end{array}$$

Then, $\dot{L}\left(x,y,v\right)<0$ is negative definite if $\hat{R}<1$. By Lyapunov's theorem, we conclude that *p*_1_=(*x*^*∗*^, 0,0) is stable on Ω.

We obtain similar results for system (4). In this case, using the Lyapunov function *L*(*x*, *w*, *y*, *v*)≔(1/2*x*^*∗*^)(*x* − *x*^*∗*^)^2^+(*pβ*/*cδ*)*x*^*∗*^(*w*+*y*)+(*β*/*c*)*x*^*∗*^*v* and Lyapunov's theorem, we prove the following result.

**Theorem 6 .**

Assume that $\hat{R}=\left(\beta \left(p-n\delta \right)/c\delta \right)x^{*}$. If $\hat{R}<1$ and *p* > *nδ*, then any infection-free equilibrium point *p*_1_=(*x*^*∗*^, 0,0,0) of line (continuum) of equilibria of system (4) is stable on Ω.

## 4. Parameter Estimation and Criterion of Model Selection

In this section, we will estimate the parameters of the models with or without the absorption effect and we will focus on model selection.

The experimental data (Table 1) provide us with observations of the total cells and viral loads, measured at ten time points *t*. We fitted the parameters simultaneously to the concentrations of total cells (*x*(*t*)+*y*(*t*) for basic model (2) and *x*(*t*)+*w*(*t*)+*y*(*t*) for latent model (4)) and viral loads (*v*(*t*)) by nonlinear least-squares regression using the function *fminsearch* of Matlab R2015a which minimizes the objective function of the sum of squared residual (SSR). We computed the SSR between the experimental data and the numerical simulation results. We also computed the second-order Akaike information criterion (AIC_C_) to compare the models with or without the absorption effect, as obtained from the following formula [12]:

(20) $$\begin{array}{c} {\text{AIC}}_{\text{C}}=N_{\text{pts}}\text{ln}\left(\frac{\text{SSR}}{N_{\text{pts}}}\right)+\frac{2\left(N_{\text{par}}+1\right)N_{\text{pts}}}{N_{\text{pts}}-N_{\text{par}}-2}, \end{array}$$

where *N*_pts_ and *N*_par_ are the numbers of data points fitted by the model and the numbers of parameters of the fitted model, respectively. The model with the lowest AIC_C_ is considered the best one. The results are shown in Table 2.

Using the criterion of model selection defined (AIC_C_), we conclude that the basic model with absorption (2) is the best.

**Figure 2:**
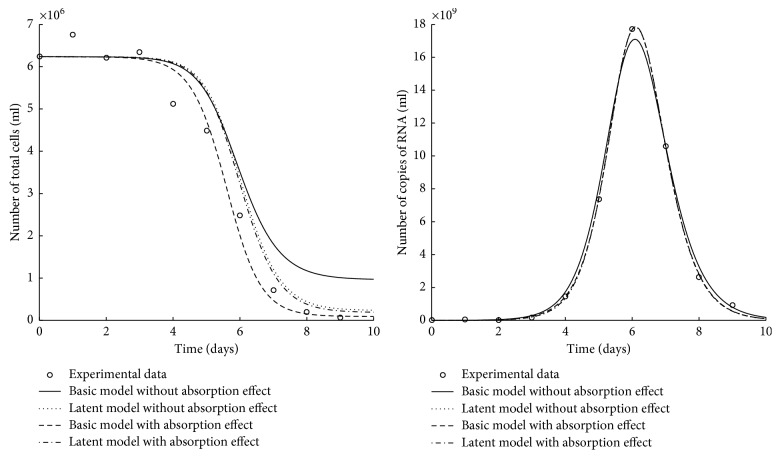
*In vitro* models fit: the best-fit line of the models with and without absorption to the experimental data (empty circles) of *SHIV* − #64 infection *in vitro* for the total cells and the viral loads.

Figure 2 shows the best fit of the *in vitro* models to the experimental data (total cells and viral loads) on the infection of HSC-F cells with *SHIV* − #64.

In addition to estimating the parameters of the models, it is also relevant to compute other amounts related to a viral infection such as the time to maximum viral load, maximum viral load, final number of target cells, area under the viral load curve (AUC), and empirical-reproductive number (*R*_e_). The results are shown in Table 3.

We observed that four models capture the maximum time of the viral load but only the basic model with absorption appropriately describes the final number of total cells. All the models describe the maximum viral load of the data with the exception of the basic model without absorption. We also noticed that the number of total cells decreases faster in the basic model with absorption. The AUC of the basic model without absorption is greater than the other models. Finally, the empirical-reproductive number (*R*_e_) was calculated for four models. We note that *R*_e_ of the basic model without absorption is approximately half that of the basic model with absorption and *R*_e_ of the latent models without or with absorption are approximately equal.

## 5. Conserved Quantity

In this section, we calculate the normalized conserved quantity (*Q*) of viral dynamics for each of the postinoculation times using the experimental viral RNA data and the estimated parameters are given in Table 2.

The equation of the normalized conserved quantity is in the terms of amounts of target cells, infected cells, and viral load, and since the experimental data are from total cells and viral load, we have to rewrite the first integral as follows:

(21) $$\begin{array}{c} Q=\frac{p}{\left(p-n\delta \right)x\left(0\right)}\left[x\left(t\right)+y\left(t\right)+w\left(t\right)+\frac{c\delta }{p}\int_{0}^{t}v\left(\tau \right)d\tau +\frac{\delta }{p}\left(v\left(t\right)-v\left(0\right)\right)-\frac{n\delta }{p}x\left(t\right)\right], \end{array}$$

For the latent model, the first three linear terms of the brackets represent the total number of cells. For the basic model, the first two linear terms represent the total number of cells. The last term is estimated with the following function *x*(*t*)=*x*(0)exp(−*β*∫_0_^*t*^*v*(*τ*)*dτ*). Finally, the area under the curve of viral load (∫_0_^*t*^*v*(*τ*)*dτ*) is calculated with the Matlab function *trapz*.

**Figure 3:**
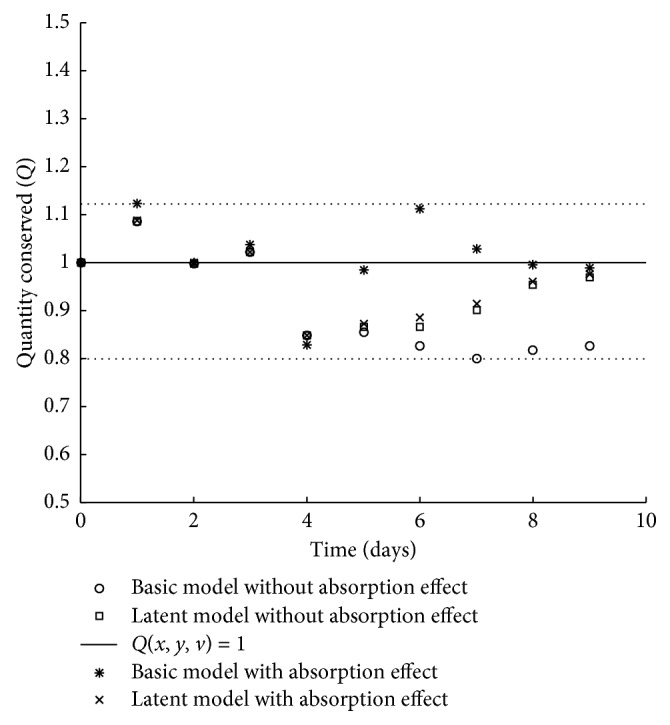
Normalized conservation quantity of the four models.

Figure 3 shows the values of normalized conserved quantity of models. The solid line segment represents the theoretical value of *Q*(*x*, *y*, *v*)=1, assuming that the experiment was carried out under ideal conditions. The values for this quantity are presented in Table 4.

Finally, we contrasted the values of conserved quantity using the one-sample Student's *t*-test under the null hypothesis: *Q*(*x*, *y*, *v*)=1 (Table 4). The value of *p* for the basic model without absorption (*p*  value=0.022) was statistically significant; we have statistical evidence to conclude that the conserved quantity is different from the unit. The value of *p* for the latent model without absorption (*p*  value=0.078) tended to be significant. On the other hand, for the models with absorption, we concluded that we do not reject the null hypothesis (*p*  value=0.714 and *p*  value=0.102).

## 6. Numerical Simulations

In this section, we use numerical simulations to visualize some qualitative properties of the trajectories of *in vitro* virus model (2) with absorption.

Theorem 3.5 gives a condition on the parameters for the stability of any infection-free equilibrium point of line (continuum) of equilibria *p*_1_=(*x*^*∗*^, 0,0) with *x*^*∗*^ > 0. In the following, we complement these theoretical results with some numerical simulations.

**Figure 4:**
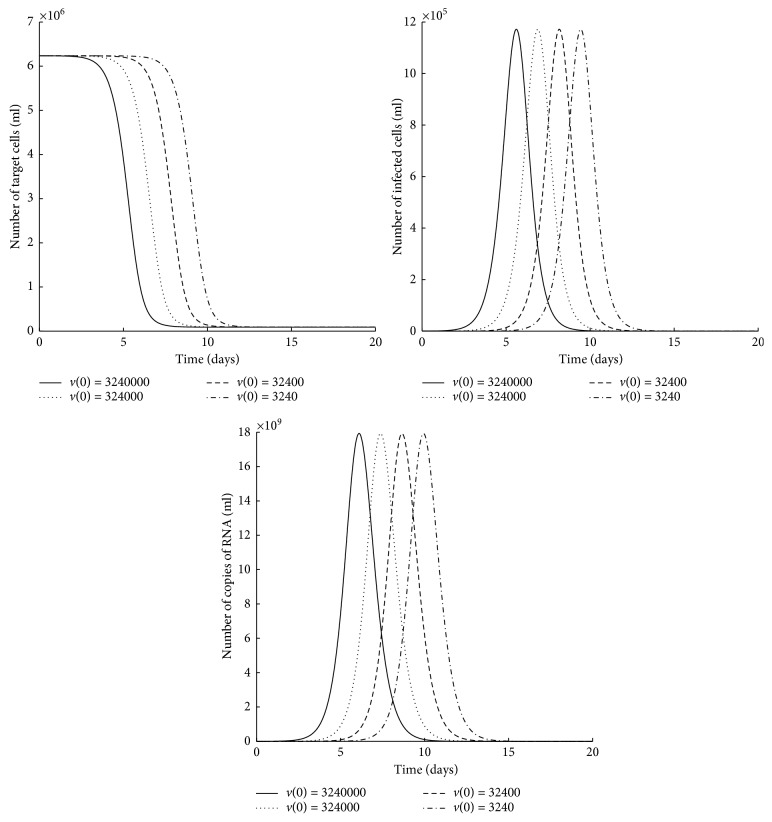
Time plots for *in vitro* virus infection model (2) for different initial conditions of viral load. The parameter values are as in Table 2, and other initial conditions are *x*(0)=6235000 and *y*(0)=0.

Figure 4 indicates that, for different initial conditions of viral load, the solutions converge at different infection-free equilibrium points ((3636,0,0), (64226,0,0), (86549,0,0), and (89235,0,0)) of infinitesimal equilibria *p*_1_. The condition $\hat{R}=\left(\beta \left(p-n\delta \right)/c\delta \right)x^{*}<1$ is satisfied for each equilibrium point.

**Figure 5:**
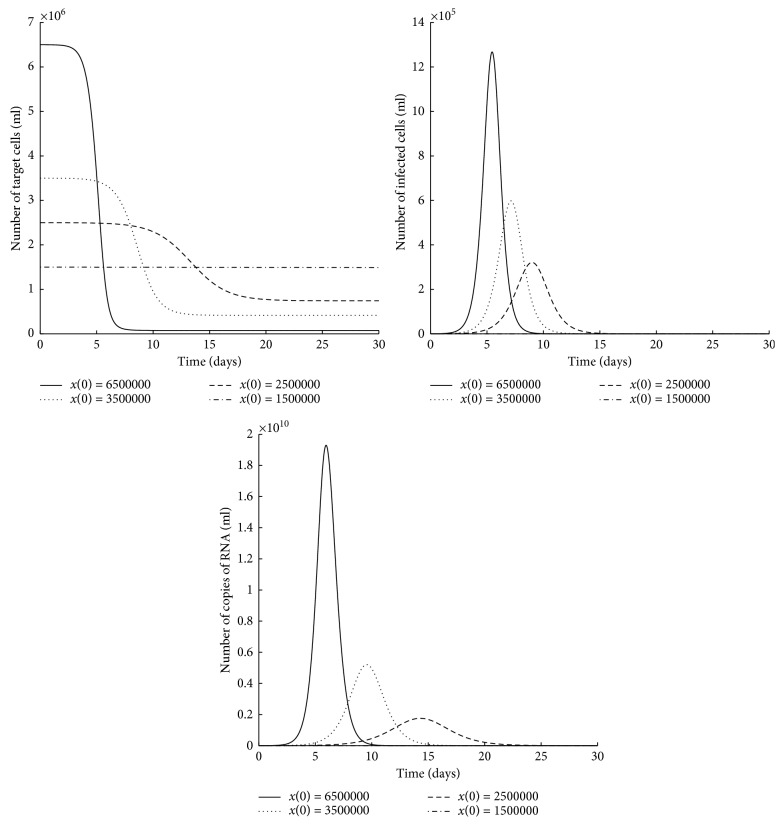
Time plots for *in vitro* virus infection model (2) for different initial conditions of the number of target cells. The parameter values are as in Table 2, and other initial conditions are *y*(0)=0 and *v*(0)=3240000.

Figure 5 indicates that, for different initial conditions of the number of target cells, similarly, the trajectories converge at different infection-free equilibrium points ((3157,0,0), (13549,0,0), (19330,0,0), and (23159,0,0)) of infinitesimal equilibria *p*_1_. The condition $\hat{R}<1$ is satisfied for each equilibrium point.

The above numerical simulations show the existence of multiple stable equilibrium points for different initial conditions with a given set of parameters.

## 7. Concluding Remarks

We studied two models of an *in vitro* viral infection that incorporated the absorption effect of viral particles. Systems (2) and (4) admitted a conserved quantity, which we interpreted as the total biological material (amino acids, nucleic acids, and other biomolecules) is preserved from the start to end of the viral infection.

We estimated the parameters of the basic and latent models with and without absorption by means of the method of least squares between experimental data and numerical solution. These data are obtained in [7] from cell cultures by infection of HSC-F cells with *SHIV* − #64. Using the values of *AIC*_*C*_ for the selection of models, we determined that the best model is the basic model with absorption effect. In addition, we reported that the normalized conserved quantity (*Q*(*x*, *y*, *v*)=1) of this model is statistically equal to the unity. In contrast, for the other models, there is not enough statistical evidence that the conserved quantity *Q* is equal to the unit.

Finally, we established the conditions for the stability of the basic and latent models with absorption effect by using the method of Lyapunov functions, and we showed these results through numerical simulations to the basic model.

## Figures and Tables

**Table 1:** *In vitro* experimental data on the infection of HSC-F cells with *SHIV* − #64.

Time (days)	Total cells^*∗*^ (cells·ml^−1^ × 10^4^)	Viral loads (RNA copies·ml^−1^ × 10^6^)
0	623.5	3.24
1	676.4	65.1
2	621.0	29.6
3	634.14	159
4	512.6	1460
5	448.0	7380
6	247.9	17700
7	71.4	10600
8	19.55	2620
9	5.92	917

^*∗*^Sum of target and infected cells. Data are taken from [7].

**Table 2:** Parameter estimation and criterion of model selection of the models with and without absorption.

	Unit	Basic model without absorption	Latent model without absorption	Basic model with absorption	Latent model with absorption
*β* × 10^−11^	RNA copies^−1^·ml·day^−1^	4.43	8.06	10.35	8.60
*δ*	day^−1^	2.82	3.28	2.52	3.79
*p* × 10^4^	cell^−1^·RNA copies·day^−1^	11.87	20.96	5.46	18.98
*c*	day^−1^	5.27	9.40	2.31	7.30
*n*	cell^−1^·RNA copies	NA	NA	6257.24	789.70
*ϕ*	day^−1^	NA	2.52	NA	2.28
SSR		≈1.5157 × 10^18^	≈1.7827 × 10^17^	≈1.7208 × 10^17^	≈1.6925 × 10^17^
AIC_C_		420.5982	414.1950	413.8412	443.6760

NA: Not applicable.

**Table 3:** Numerical and analytical solution of the infection-related quantities derived from the models with and without absorption.

	Unit	Basic model without absorption	Latent model without absorption	Basic model with absorption	Latent model with absorption
Time to maximum viral load	day	6.08	6.12	6.08	6.08
Maximum viral load	RNA copies·ml^−1^	1.7097 × 10^10^	1.7824 × 10^10^	1.7851 × 10^10^	1.7836 × 10^10^
Final number of target cells	cells·ml^−1^	9.6660 × 10^5^	2.3251 × 10^5^	8.9464 × 10^4^	1.8591 × 10^5^
Area under the viral load curve	RNA copies·ml^−1^	4.1516 × 10^10^	4.0474 × 10^10^	4.0663 × 10^10^	4.0520 × 10^10^
*R*_e_=(*β*(*p* − *nδ*)/*cδ*)*x*(0)		2.2098	3.4100	4.2925	3.6118

**Table 4:** Normalized conserved quantity for each of the postinoculation time periods.

Conserved quantity	*t* = 0	*t* = 1	*t* = 2	*t* = 3	*t* = 4	*t* = 5	*t* = 6	*t* = 7	*t* = 8	*t* = 9	*p*-value^*∗*^
Basic model without absorption	1.0000	1.0858	0.9977	1.0212	0.8475	0.8552	0.8254	0.7994	0.8185	0.8257	0.022
Latent model without absorption	1.0000	1.0858	0.9979	1.0216	0.8491	0.8649	0.8667	0.9005	0.9537	0.9694	0.078
Basic model with absorption	1.0000	1.1222	1.0000	1.0372	0.8281	0.9850	1.1113	1.0283	0.9945	0.9898	0.714
Latent model with absorption	1.0000	1.0872	0.9980	1.0222	0.8487	0.8727	0.8855	0.9141	0.9615	0.9759	0.102

^*∗*^Null hypothesis: *Q*(*x*, *y*, *v*)=1.

## Data Availability

Previously reported experimental data were used to support this study and are available at (DOI: 10.1016/j.jtbi.2015.03.034). These datasets are cited at relevant places within the text as Reference [7]. In addition, we included the experimental data within the paper.
